# Co-Assembly of Graphene Oxide and Albumin/Photosensitizer Nanohybrids towards Enhanced Photodynamic Therapy

**DOI:** 10.3390/polym8050181

**Published:** 2016-05-04

**Authors:** Ruirui Xing, Tifeng Jiao, Yamei Liu, Kai Ma, Qianli Zou, Guanghui Ma, Xuehai Yan

**Affiliations:** 1 State Key Laboratory of Metastable Materials Science and Technology, Yanshan University, Qinhuangdao 066004, China; rrxing@ipe.ac.cn (R.X.); liuym@ipe.ac.cn (Y.L.); makai@ipe.ac.cn (K.M.); 2 Hebei Key Laboratory of Applied Chemistry, School of Environmental and Chemical Engineering, Yanshan University, Qinhuangdao 066004, China; 3 National Key Laboratory of Biochemical Engineering, Institute of Process Engineering, Chinese Academy of Sciences, Beijing 100190, China; ghma@ipe.ac.cn; 4 Center for Mesoscience, Institute of Process Engineering, Chinese Academy of Sciences, Beijing 100190, China

**Keywords:** protein, graphene oxide, singlet oxygen generation, co-assembly, photodynamic therapy

## Abstract

The inactivation of photosensitizers before they reach the targeted tissues can be an important factor, which limits the efficacy of photodynamic therapy (PDT). Here, we developed co-assembled nanohybrids of graphene oxide (GO) and albumin/photosensitizer that have a potential for protecting the photosensitizers from the environment and releasing them in targeted sites, allowing for an enhanced PDT. The nanohybrids were prepared by loading the pre-assembled nanoparticles of chlorin e6 (Ce6) and bovine serum albumin (BSA) on GO via non-covalent interactions. The protection to Ce6 is evident from the inhibited fluorescence and singlet oxygen generation activities of Ce6–BSA–GO nanohybrids. Importantly, compared to free Ce6 and Ce6 directly loaded by GO (Ce6–GO), Ce6–BSA–GO nanohybrids showed enhanced cellular uptake and *in vitro* release of Ce6, leading to an improved PDT efficiency. These results indicate that the smart photosensitizer delivery system constructed by co-assembly of GO and albumin is promising to improve the stability, biocompatibility, and efficiency of PDT.

## 1. Introduction

Photodynamic therapy (PDT) is attractive for cancer therapy due to its specific selectivity to a disease site and non-invasive protocol [1,2]. Essentially, PDT involves the administration of a photoactive drug (photosensitizer), followed by selective irradiation of the cancerous tissue by light [3]. The activated photosensitizer reacts with molecular oxygen, resulting in the formation of reactive oxygen species (ROS), such as singlet oxygen, that are directly responsible for the death of cancer cells [4]. Hence, the cytotoxicity in PDT is a combined result of three nontoxic components (photosensitizer, molecular oxygen, and light), leading PDT to be a highly-biocompatible treatment modality for cancer. Despite the fast-growing research about PDT [5,6,7,8,9,10,11], there are still huge challenges before its acceptance as a first-line oncological treatment. Due to the limited selective accumulation of photosensitizers to tumors [12], a large amount of photosensitizers are needed to obtain a satisfactory photodynamic response. Importantly, it typically takes 48–72 h for the photosensitizers to accumulate in tumors, leading to the unprotected photosensitizers vulnerable in the circulatory system [13]. An intriguing method for circumventing these challenges is to formulate the photosensitizers in means of suitable nanocarriers [13,14,15,16]. The nanocarriers can be tailored to protect the encapsulated photosensitizers in circulation and readily release them in targeted tissues.

Due to its special photochemical properties, graphene has attracted remarkable attention in many fields including smart drug delivery [17,18,19]. The planar and highly-conjugated morphology of graphene sheet is ideally suitable for loading of drugs through the non-covalent interactions, such as hydrophobic interaction and π–π stacking [20]. The unique two-dimensional structure of graphene sheet ensures a high loading capability for physical adsorption of drugs on its large surface [21]. Graphene oxide (GO), a derivative of graphene, not only has the same merits as graphene but also holds better solubility and more functional groups suitable for further modification [22,23]. GO can be converted to nano-GO with the lateral dimensions in nanometric size by applying ultrasonic energy [24,25]. Nano-GO has been explored as a nanocarrier for delivery of various drugs including photosensitizers [26]. It has been reported that the photosensitizers loaded onto nano-GO can be more efficiently delivered into cells [27,28]. Since most photosensitizers are highly π-conjugated and, among hydrophobic porphyrin derivatives, the loading of them on the surface of GO can enhance their aqueous solubility and stability. Nano-formulation can further improve the accumulation of photosensitizers to tumors through the enhanced permeability and retention (EPR) effect [29]. However, the highly efficient π–π stacking between the photosensitizers and GO inhibits the ROS generation ability of the photosensitizers [30]. Since GO is highly stable in biological environments [31], release of photosensitizers through passive biodegrading of GO is inefficient. Consequently, the release of the photosensitizers from the surface of GO with a specific design is needed. To the best of our knowledge, such a GO-based nano-formulation of photosensitizers with specific loading and release properties has not yet been explored.

Serum albumin, the most abundant protein in blood plasma, has been approved by FDA for drug delivery based on its high biocompatibility [32]. The encapsulation of various hydrophobic drugs by serum albumin has been extensively studied [33,34]. It has been demonstrated that albumins are highly promising in encapsulation and release of photosensitizers [35]. As most nanoparticles are internalized by cells through a lysosome-mediated channel, the lysosomal cysteine proteases, such as cathepsin B, could induce the intracellular biodegradation of albumin [36,37], triggering the release of encapsulated photosensitizers. Therefore, combination of albumin with other functional materials, such as GO, by the strategy of nanoarchitectonics may be a versatile means for delivery of photoactive drugs towards enhanced PDT [38,39,40].

**Scheme 1:**
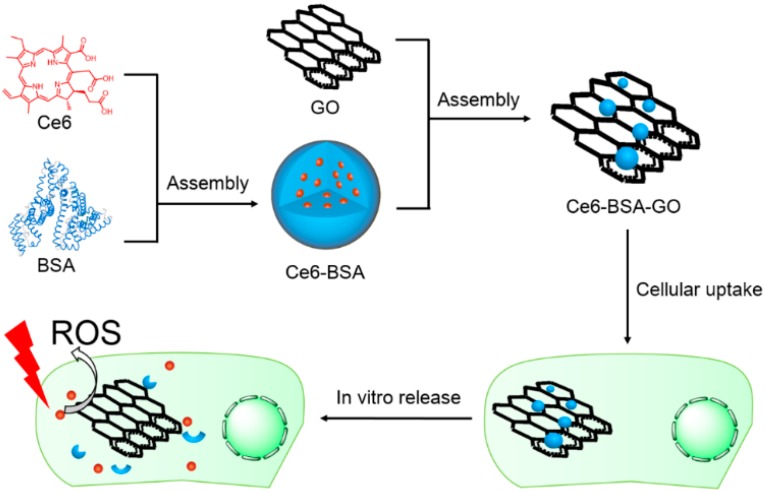
Schematic illustration of co-assembly of graphene oxide and albumin/photosensitizer nanohybrids and the enhanced *in vitro* photodynamic therapy.

Herein, we report a co-assembly strategy for the fabrication of nanohybrids of GO, albumin and photosensitizer for *in vitro* enhanced PDT (Scheme 1). In the nanohybrids, the hybrid system of albumin and GO makes synergistic effect as delivery carriers. The nanohybrids are prepared by loading of the pre-assembled nanoparticles of chlorin e6 (Ce6) and bovine serum albumin (BSA) on the surface of nano-GO. We discover that both the fluorescence and ROS of as-prepared Ce6-BSA-GO nanohybrids in physiological conditions are quenched as compared to free Ce6. Additionally, Ce6–BSA–GO nanohybrids show a much faster internalization and release of Ce6 upon incubation with cells when compared with free Ce6 or a control group of Ce6–GO (Ce6 directly loaded on the surface of GO). *In vitro* PDT results show that Ce6 released from Ce6–BSA–GO nanohybrids recovers its photocytotoxicity upon the cellular uptake. Thus, an enhanced PDT efficacy of Ce6–BSA–GO nanohybrids is demonstrated. Such nanohybrids prepared by encapsulation and loading of photosensitizer by albumin and GO provide a valuable approach to construct smart and biocompatible PDT agents on demand.

## 2. Materials and Methods

### 2.1. Materials

Bovine serum albumin, anthracene-9,10-dipropionic acid (ADPA), thiazolyl blue tetrazolium bromide (MTT), and Hoechst 33342 were purchased from Sigma-Aldrich Co. (Saint Louis, MI, USA) Chlorin e6 was purchased from Frontier Scientific, Inc. (Logan, UT, USA). The cell culture medium DMEM was purchased from Beijing Solarbio Science and Technology Co. Ltd. (Beijing, China). Fetal bovine serum (FBS) was purchased from Hangzhou Sijiqing Co. Ltd. (Hangzhou, China). Other chemicals were purchased from Beijing Chemical Co. Ltd. (Beijing, China) unless otherwise specified. All chemicals were used as received without further purification. Nano graphene oxide was synthesized according to our previous report [25]. Briefly, graphite, NaNO_3_, and concentrated H_2_SO_4_ were mixed together in a beaker in an ice bath for 30 min, followed by the slow addition of KMnO_4_. The reaction mixture was stirred at 35 °C for 6 h, and then the temperature was slowly raised to 60 °C during the next 2 h. The above mixture was then added to water and was stirred at 80 °C for 1 h, followed by adding 30% H_2_O_2_ and filtering. For purification, the product was alternately washed with 5% of HCl and then water several times. The filter cake was dissolved in water and the graphene oxide flakes were obtained through centrifugation. Finally, the product was freeze-dried in a lyophilizer for two days before use.

### 2.2. Preparation of the Nanohybrids

The Ce6–BSA–GO nanohybrids were typically prepared as follows: firstly, Ce6-BSA nanoparticles were prepared by mixing a DMSO solution of Ce6 (12 mg·mL^−1^) with an aqueous solution of BSA (4 mg·mL^−1^). After aging in the dark for 4 h, the Ce6-BSA nanoparticles were mixed with an aqueous solution of GO (0.5 mg·mL^−1^). Finally, the obtained nanohybrids were aged overnight and washed by dialysis before further characterization. A control group of Ce6-GO was prepared by mixing a DMSO solution of Ce6 (12 mg·mL^−1^) with an aqueous solution of GO (0.5 mg·mL^−1^).

### 2.3. Characterization of the Nanohybrids

Transmission electron microscopy (TEM) was performed by a JEOL JEM-2100 (Kyoto, Japan) when a drop of the sample was carefully applied to the carbon-coated copper grids and dried in vacuum. A Bruker FastScanBio was used for atomic-force microscopy (AFM) measurements. The size and zeta potential of the nanohybrids was characterized by Malvern dynamic laser scattering (DLS) (Zetasizer Nano ZS ZEN3600, Malvern, UK). UV–Vis spectra of the assembled nanohybrids in aqueous solution were recorded using a Shimadzu UV-2600 spectrophotometer (Kyoto, Japan). A fluorescence spectrometer (Hitachi F-4500, Kyoto, Japan) was used to measure the photoluminescence of free Ce6 and assembled nanohybrids in a 1.0 cm quartz cuvette with the excitation wavelength of 405 nm. The concentrations of Ce6 in samples were measured by a HPLC-based method. Briefly, the sample (100 μL) was mixed with acetonitrile (1 mL) and the obtained mixture was sonicated by an ultrasonic cell crusher (JY92-IIN, Ningbo Scientz Biotechnology Co., Ningbo, China) The concentration of Ce6 in the mixture was further recorded on a Thermo Fisher U3000 HPLC system coupled with a VWD-3100 detector and a reverse phase C18 column (Thermo Scientific Acclaim 120, 5 μm, 4.6 mm × 250 mm, product number 059149, Waltham, MA, USA). Chromatographic conditions: 25 °C; 1.0 mL·min^−1^; 405 nm; gradient solvent system: *v*/*v* acetonitrile/0.1% trifluoroacetic acid in water, and a stepwise gradient of acetonitrile from 45% acetonitrile to 100% in 20 min; *t* = 9.75 min. In the assembled nanohybrids of Ce6–BSA–GO, the loading amount of Ce6 was evaluated to be 5.5% (*w*/*w*).

### 2.4. ROS Generation

The generation of ROS was detected by the bleaching of 9,10-anthracene dipropionic acid (ADPA). A mixed solution of the nanohybrids and ADPA was prepared. During the following experiment, the solution was stirred vigorously to ensure the saturation of air. When the solution was irradiated by a 635 nm laser (10 mW·cm^−2^), the bleaching of the absorption band of ADPA at 399 nm was monitored. The solution containing only ADPA was also irradiated by the laser and set as a control group.

### 2.5. Cell Culture

HeLa cells, generously provided by Prof. Junbai Li (Institute of Chemistry, CAS, Beijing, China), were cultured in the cell culture medium (DMEM supplemented with 10% FBS) at 37 °C under 5% CO_2_ atmosphere according to standard cell culture protocols.

### 2.6. In Vitro Imaging

The cells were trypsinized and seeded in a 35 mm glass-bottom dish with a density of 5 × 10^4^ cells per well in 2 mL of culture medium. After 24 h, an aliquot of nanohybrids was added to the dish to ensure a Ce6 concentration of 10 μg·mL^−1^. At pre-determined time points, the cells were washed with phosphate-buffered saline (PBS), stained with Hoechst 33342 fluorescent DNA-binding dye at 5 μg·mL^−1^, and observed by a confocal laser scanning microscopy (CLSM, Olympus FV1000, Kyoto, Japan). Hoechst 33342 was excited at 405 nm, while Ce6 was excited at 635 nm.

### 2.7. In Vitro PDT

The cells were seeded to 96-well plates with a density of 2.5 × 10^4^ cells per well and were incubated for 24 h. Then, the cells were further incubated with the nanohybrids for 24 h. The cells were then washed with fresh culture medium and irradiated by a 635 nm laser (0.2 W·cm^−2^) for 1 min. After irradiation, the cells were incubated for another 24 h before the cell viability test by the MTT assay according to the manufacture’s protocol.

## 3. Results and Discussion

### 3.1. Preparation and Characterization

**Figure 1:**
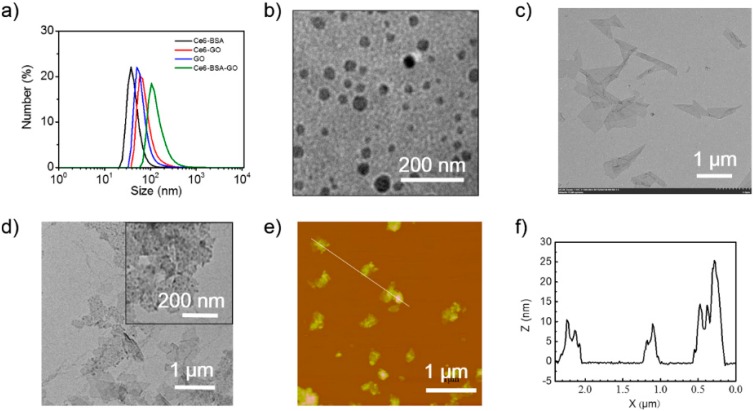
The size and morphology of the nanohybrids. (**a**) Size distribution of Ce6–BSA, Ce6–GO, GO, and Ce6–BSA–GO; (**b**) TEM image of Ce6–BSA; (**c**) TEM image of GO; (**d**) TEM images of Ce6–BSA–GO; (**e**) AFM image of Ce6–BSA–GO; and (**f**) the line profile of Ce6–BSA–GO obtained from (**e**).

Ce6 selected for this study is a representative second-generation photosensitizer for PDT [41]. Due to its large molar extinction coefficient in the near-infrared range, Ce6 has been extensively studied for PDT [42]. However, free Ce6 aggregates easily in aqueous solution, disenabling the direct application of free Ce6 for PDT. Ce6–BSA–GO nanohybrids were prepared via two steps, including the preparation of Ce6-BSA nanoparticles and the loading of Ce6–BSA nanoparticles by nano-GO. Ce6–BSA nanoparticles were prepared by co-assembly of Ce6 and BSA. The DLS size distribution shows that Ce6–BSA nanoparticles have a mean hydrodynamic diameter of 38 ± 10 nm (Figure 1a). TEM image of Ce6–BSA nanoparticles (Figure 1b) indicates that they possess a spherical morphology and their average diameter is almost in agreement with the DLS result. By mixing the pre-assembled Ce6-BSA nanoparticles with nano-GO, negatively-charged Ce6–BSA–GO nanohybrids (zeta potential: −18 ± 5.6 mV) with a mean hydrodynamic diameter of 112 ± 40 nm were obtained. Since nano-GO has a mean hydrodynamic diameter of 53 ± 14 nm, the size increment after mixing suggests the successful loading of Ce6-BSA nanoparticles. The size of graphene oxide is on the scale of 1000 nm, as measured by TEM (Figure 1c), but less than 100 nm in hydrodynamic diameter detected by DLS in solutions. This is because samples are dried in vacuum before characterization by TEM. In such a dry state, GO is in the shape of a film, whereas GO tends to curl in the aqueous solution. That is why the sizes of GO and Ce6–BSA–GO characterized by TEM are much larger than the ones obtained by DLS. TEM images of nano-GO (Figure 1c) and Ce6–BSA–GO (Figure 1d) also confirm that Ce6–BSA nanoparticles are loaded on the surface of nano-GO. AFM image (Figure 1e) and topographic height diagram (Figure 1f) of Ce6–BSA–GO nanohybrids show a height of 10–30 nm, also consistent with the size of Ce6-BSA nanoparticles. The loading of Ce6-BSA on the surface of GO should be the result of hydrophobic and π–π stacking interactions between GO and aromatic residues of BSA as revealed by recent studies [43,44]. Meanwhile, it has been demonstrated that such non-covalent interactions between BSA and GO is strong in a physiological condition of 10 mM phosphate buffer solution at pH 7.4 [43]. Hence, Ce6–BSA–GO nanohybrids are expected to be suitable for drug delivery. For comparison, Ce6–GO nanohybrids with a mean hydrodynamic diameter of 38 ± 10 nm (Figure 1a) were prepared by directly loading of Ce6 on GO.

**Figure 2:**
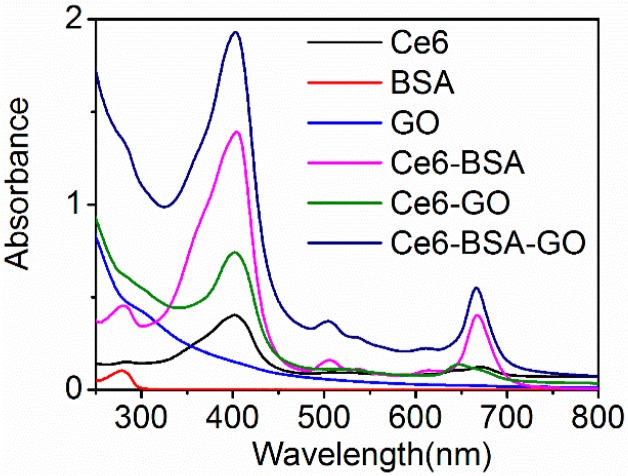
UV–Vis absorption spectra of Ce6, BSA, GO, Ce6-BSA, Ce6-GO, and Ce6–BSA–GO.

UV–Vis absorption spectra of Ce6–BSA–GO nanohybrids, the starting components, and the intermediate materials (Figure 2) were studied to reveal the aggregation status of Ce6 in the nanohybrids. Free Ce6 in water shows a decreased Q-band at 670 nm due to the aggregation induced by intermolecular hydrophobic and π–π stacking interactions [45]. The presence of a characteristic peak at 280 nm of BSA in Ce6–BSA confirms the presence of BSA in the formed Ce6–BSA nanoparticles. Importantly, Ce6–BSA nanoparticles show an increased Q-band, indicating that the aggregation of Ce6 is partly inhibited by the interactions between Ce6 and BSA. This may attribute to the encapsulation of Ce6 in the hydrophobic domains of BSA [46,47]. The absorption bands of Ce6–BSA–GO are similar to those of Ce6–BSA, suggesting the non-covalent interactions between BSA and GO have no significant impact on the absorption of Ce6. By contrast, Ce6–GO shows a significant decreased Q-band, suggesting that a higher degree of aggregation of Ce6 may be induced by the direct contact with GO.

### 3.2. Fluorescence and ROS

**Figure 3:**
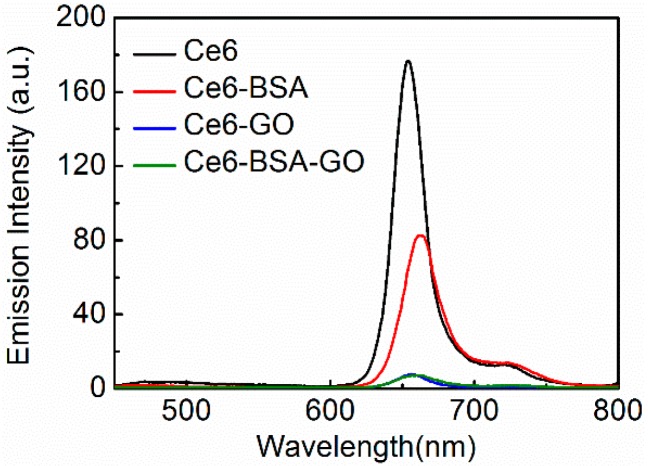
Fluorescence emission spectra of Ce6, Ce6–BSA, Ce6–GO, GO, and Ce6–BSA–GO. The samples were adjusted to contain the same amount of Ce6 and excited at 405 nm.

In PDT, the generation of cytotoxic ROS is induced by the irradiation. As the light irradiation will be applied to the selected area for a limited time, it is more favorable to inhibit the ROS generation of the photosensitizer before it can be accumulated in the targeted tissue. At present, clinically-applied photosensitizers are always sensitive to light. Hence, special protections are needed in storage of these photosensitizers and the patients must be kept from light for a long time [13]. To examine whether the nanohybrids can inhibit the ROS activity of Ce6, a fluorescence assay was conducted because fluorescence and ROS share the same pathway of excitation and they could be quenched by aggregation simultaneously. In the fluorescence experiment, the emission spectra of Ce6–BSA–GO were compared with various samples containing the same amount of Ce6 (Figure 3). The emission intensities of Ce6–BSA–GO and Ce6–GO are almost identical and are found to be significantly quenched in comparison with those of Ce6 and Ce6–BSA. Although the absorption spectra suggest a low aggregation degree of Ce6 in Ce6–BSA, the emission intensity of Ce6–BSA is found lower than that of Ce6, suggesting that the energy transfer occurs between Ce6 and BSA [4]. In addition to the energy transfer between Ce6 and BSA, an additional energy transfer between Ce6 and GO is also presumably responsible for the low florescence intensity of Ce6–BSA–GO, along with the self-quenching of Ce6.

**Figure 4:**
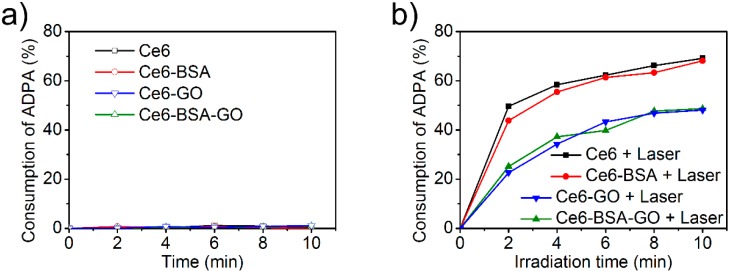
Cumulative consumption of ADPA by the ROS generated by Ce6, Ce6–BSA, Ce6–GO, and Ce6–BSA–GO in the (**a**) absence and (**b**) presence of irradiation.

To monitor the ROS activity of Ce6–BSA–GO directly, ADPA was applied as a ROS-sensitive sensor. ADPA is a water-soluble π-conjugated sensor with a characteristic absorption band at 400 nm [48]. In the presence of ROS, the band at 400 nm decreases due to the breaking of the conjugation. The experiments were conducted by irradiation of the samples containing the same concentration of Ce6 and ADPA. At various time terminals, the absorbance at 400 nm was monitored. When the samples were kept in the dark, no ROS was detected (Figure 4a). When irradiated, Ce6–BSA–GO generated similar amounts of ROS with Ce6–GO, while both of them showed lower ROS activities than Ce6 and Ce6–BSA (Figure 4b). This result is highly consistent with the changes in florescence intensity, indicating the energy transfer between Ce6 and GO inhibits the generation of both fluorescence and ROS. It should be noted that the ROS activity of free Ce6 is partially inhibited due to the self-aggregation. The further inhibited ROS activity of Ce6–BSA–GO enables a lower sensitivity and a higher stability before the release of Ce6.

### 3.3. Cellular Uptake and In Vitro Release

**Figure 5:**
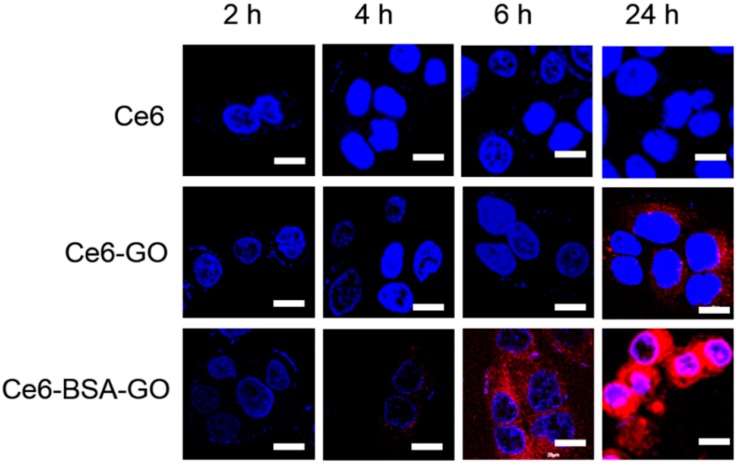
Confocal images of HeLa cells incubated with Ce6, Ce6–GO, or Ce6–BSA–GO for various times and stained by Hoechst 33342. Scale bars denote 15 μm.

The ROS, especially singlet oxygen, have a short lifetime and a small diffusion distance in an aqueous environment. As a consequence, the efficient internalization by the cells is crucial for the nanohybrids to achieve a better therapeutic efficiency. In observation of the inhibited ROS activity of Ce6–BSA–GO, we next investigated the cellular internalization and release of Ce6–BSA–GO. CLSM images of the HeLa cells incubated with the nanohybrids for diverse times and stained with Hoechst 33342 show that Ce6–BSA–GO is efficiently internalized by cells and the release of Ce6 from Ce6–BSA–GO is efficiently triggered by cellular microenvironment (Figure 5). Only the fluorescence of Hoechst 33342 is observed in the cells incubated with Ce6 for 24 h. By contrast, the fluorescence of Ce6 is shown in the cytoplasm of the cells incubated with Ce6–GO for 24 h, indicating that the cellular internalization of Ce6–GO is more efficient than free Ce6, which is consistent with a previous report [30].

For the cells incubated with Ce6–BSA–GO, the fluorescence of Ce6 is obviously shown at 6 h of incubation and significantly enhanced at 24 h of incubation. Significantly, the cells incubated with Ce6–BSA–GO show higher fluorescence intensity than those incubated with Ce6–GO, implying that the Ce6 is more readily released from Ce6–BSA–GO, presumably due to the enzymatic degradation of BSA in lysosomes [49]. The enhanced uptake and release activity of Ce6–BSA–GO allow them to quickly recover the ROS generation ability once accumulated in tumors.

### 3.4. In Vitro PDT

**Figure 6:**
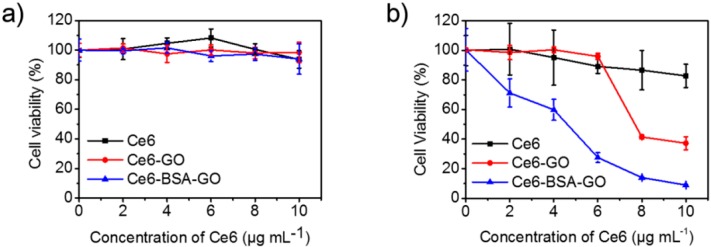
*In vitro* PDT. (**a**) MTT cell viability of HeLa cells incubated with the nanohybrids in dark for 24 h; (**b**) MTT cell viability of HeLa cells incubated with the nanohybrids in dark for 24 h, followed by the irradiation by a 635 nm laser (0.2 W·cm^−2^) for 1 min.

One advantage of PDT is that the photosensitizers, themselves, are non-toxic to cells in the absence of light. To verify the biocompatibility of the nanohybrids for cells, we incubated HeLa cells with a variety of concentration of nanohybrids in dark for 24 h and checked the cell viabilities by MTT assays. The results show that the cell viabilities are not affected by Ce6, Ce6–GO, or Ce6–BSA–GO at a Ce6 dosage up to 10 μg·mL^−^^1^ (Figure 6a), indicating the nanohybrids are biocompatible for PDT. To further investigate the photocytotoxicity of the nanohybrids, HeLa cells were incubated with Ce6, Ce6–GO, and Ce6–BSA–GO at a series of Ce6 concentrations for 24 h, followed by irradiation with a 635 nm laser (0.2 W·cm^−^^2^) for 1 min. The cell viabilities were determined by MTT assays at 24 h of post-treatment (Figure 6b). The results show that the cell viability was not affected only by light. When samples containing Ce6 were added, the photocytotoxicity started to occur and increased with the increasing Ce6 dosage. Ce6–GO shows a higher photocytotoxicity when compared with Ce6, consistent with the results of *in vitro* fluorescence intensity. The IC50 (the concentration of a photosensitizer inhibits 50% of the cells under light) values of Ce6–BSA–GO and Ce6–GO are 4.5 and 7.8 μg·mL^−1^, respectively. The lower IC50 of Ce6–BSA–GO than Ce6–GO implies that more efficient release of Ce6 and, consequently, a higher degree of ROS recovery are realized by the application of BSA. The viability of cells pretreated by Ce6–BSA–GO at a Ce6 dosage of 10 μg mL^−^^1^, followed by irradiation, decreases to 8.95%. This suggests that a high PDT efficacy is successfully obtained by the nanohybrids co-assembled from photosensitizer, albumin, and GO.

## 4. Conclusions

We have demonstrated a smart drug delivery system for photosensitizers by using GO as a ROS quencher and BSA as a biologically-derived degradable component for accelerating the intracellular release. The formed Ce6–BSA–GO nanohybrids show inhibited fluorescence and ROS activities compared to free Ce6 and Ce6-GO. The low ROS activity is beneficial for the protection of a photosensitizer before its accumulation in targeted tissues, such as in storage and in the circulation system. We also demonstrate that the fluorescence and ROS activities of Ce6–BSA–GO are efficiently recovered once they are internalized by cancer cells. The cells incubated with Ce6–BSA–GO are photosensitive and show a more effective PDT that the cells incubated with free Ce6 or Ce6–GO. This proof-of-concept drug delivery system, combining the advantages of binary components of GO and BSA, has the potential to be developed as a robust and versatile tool for delivery of a wide range of photosensitive drugs.

## Abbreviations

The following abbreviations are used in this manuscript:

PDT	Photodynamic therapy
GO	Graphene oxide
Ce6	Chlorin e6
BSA	Bovine serum albumin
ROS	Reactive oxygen species
EPR	Enhanced permeability and retention
MTT	Thiazolyl blue tetrazolium bromide
FBS	Fetal bovine serum
TEM	Transmission electron microscope
AFM	Atomic-force microscope
DLS	Dynamic laser scattering
ADPA	9,10-anthracene dipropionic acid
PBS	Phosphate buffered saline
CLSM	Confocal laser scanning microscopy
